# Thrombotic Risk and Hemostatic Profiles in Pediatric Inflammatory Bowel Disease: Association with Disease Activity

**DOI:** 10.5152/tjg.2026.25541

**Published:** 2026-03-24

**Authors:** Yasin Maruf Ergen, Edibe Gözde Başaran, Selçuk Teke, Tuğba Çaviş, Necati Balamtekin

**Affiliations:** 1 Division of Pediatric Gastroenterology, Department of Pediatrics, University of Health Sciences, Gülhane Training and Research Hospital, Ankara, Türkiye; 2 Department of Radiology, University of Health Sciences, Gülhane Training and Research Hospital, Ankara, Türkiye

**Keywords:** Blood coagulation, factor VIII, inflammation, inflammatory bowel diseases, venous thrombosis

## Abstract

**Background/Aims:**

Inflammatory bowel disease (IBD) increases thromboembolic risk, but pediatric evidence remains limited. The current study evaluated the associations between coagulation markers, systemic inflammation, and disease activity in pediatric IBD and assessed the prevalence of silent thrombosis.

**Materials and Methods:**

A total of 46 pediatric patients with IBD were enrolled in this single-center cross-sectional study. All underwent Doppler ultrasonography of the lower extremities and the portal venous system. Hemostatic and inflammatory markers were evaluated in the laboratory and were analyzed in relation to disease activity.

**Results:**

One asymptomatic thrombus was detected in a patient with Crohn’s disease (CD). Factor VIII was significantly higher in ulcerative colitis (UC) compared to CD (median 109.5 vs. 74.3 IU/dL, *P* = .007). In UC, platelet count and D-dimer increased with increasing disease activity and extent. There was a significant relationship between platelet counts and the endoscopic extent (*P* = .008) and Mayo Score (*P* = .031). In CD, fibrinogen and factor VIII levels revealed a positive correlation with Pediatric Crohn’s Disease Activity Index scores (*P* = .012 and *P* = .040, respectively) but not with the Simple Endoscopic Score. Corticosteroid use was linked to higher factor VII (*P* = .017).

**Conclusion:**

Asymptomatic thrombosis rare in IBD, but inflammation and procoagulant activity were closely related, especially in UC. Platelet count, D-dimer, and factor VIII might serve as biomarkers for thrombotic risk and monitoring.

**Main Points**

- Although clinical thrombosis is rare in pediatric inflammatory bowel disease (IBD), alterations in hemostatic parameters may indicate a prothrombotic state.
- Platelet count, D-dimer, and factor VIII levels strongly correlate with inflammation and disease activity, particularly in ulcerative colitis.
- Standard hemostatic markers may support disease monitoring and risk stratification when invasive procedures are not feasible.
- Routine screening for silent thrombosis appears to have low diagnostic yield in patients with asymptomatic pediatric IBD.
- Future multicenter studies are needed to define specific cut-off values for coagulation markers in pediatric clinical practice.

## Introduction

Venous thromboembolism (VTE) is a rare but potentially life-threatening complication of inflammatory bowel disease (IBD). Pediatric studies reported an incidence of 5.5-31.2 per 10 000 patient-years, highlighting the need for vigilance.^1^^,^^2^ Inflammation promotes thrombosis by upregulating coagulation factors, inhibiting fibrinolysis, and reducing natural anticoagulants. In adults with IBD, procoagulant changes such as elevated fibrinogen, von Willebrand factor (vWF), and clotting factors V and VIII, together with reduced protein C, protein S, and antithrombin III (AT III), have been consistently reported. These alterations indicate the inflammatory response and may occur regardless of clinically apparent VTE.^3^

Recent large-scale multicenter studies have documented the increasing prevalence and evolving epidemiological landscape of IBD.^4^ However, pediatric evidence remains more limited and heterogeneous compared to the adult literature. While adult studies have linked specific markers—such as elevated factor VIII and fibrinogen—to active disease and thromboembolic risk, pediatric reports have shown less consistent findings. Furthermore, most pediatric studies have primarily relied on clinical activity indices without concurrent objective assessment of mucosal inflammation, leaving an important gap in understanding how hemostatic parameters correlate with endoscopic severity and extent. Consequently, there is a lack of validated pediatric thresholds or biomarkers to help guide screening and prophylaxis decisions in children.^5^

The primary objective of the present study was to investigate the associations between hemostatic parameters and disease activities in children with IBD. As a secondary objective, the prevalence of asymptomatic thrombosis was evaluated, and the influence of therapeutic regimens was explored.

## Materials and Methods

### Study Design and Participants

This was a single-center cross-sectional study conducted between May and July 2025 at the tertiary pediatric gastroenterology clinic of Gülhane Training and Research Hospital. Patients who were aged between 6 and 18 years and who had a confirmed diagnosis of ulcerative colitis (UC) or Crohn’s disease (CD) according to European Society for Paediatric Gastroenterology, Hepatology and Nutrition (ESPGHAN) criteria were were included to the study.^6^^-^^8^ Either newly diagnosed patients undergoing diagnostic endoscopy or follow-up patients for whom endoscopic evaluation was clinically indicated to assess disease activity, mucosal healing status, or treatment response were eligible for inclusion in the study. During the same visit, clinical assessment and blood sampling were carried out to ensure standardized data collection.

Exclusion criteria included unrelated systemic diseases, liver disorders, recent blood transfusion, known coagulopathies, thrombophilia in a first-degree relative, presence of central venous catheter, or active infection. Children experiencing acute severe exacerbations requiring hospitalization were not included to avoid potential confounding effects on coagulation parameters.

### Thrombosis Screening

All patients underwent Doppler ultrasonography of the lower extremity deep venous system (common femoral, superficial femoral, popliteal, and iliac veins) as well as the portal venous system to detect asymptomatic thrombosis. Examinations were performed during the same visit when blood sampling and endoscopic evaluation was done. Ultrasonography was performed by an experienced radiologist using a standardized protocol, and the examiner was blinded to the patient’s clinical and laboratory data.

### Clinical and Endoscopic Assessment

Clinical disease activity in UC was assessed using the Pediatric Ulcerative Colitis Activity Index (PUCAI).^9^ Disease severity was evaluated more comprehensively using the Total Mayo Score and mucosal inflammation was specifically graded using the Endoscopic Mayo Score (ranging from 0 to 3).^10^ The endoscopic disease extent was classified according to the Paris criteria: E1, proctitis (limited to the rectum); E2, left-sided colitis (up to the splenic flexure); E3, extensive colitis (up to the hepatic flexure); and E4, pancolitis (involving the entire colon).^11^ Patients with previously diagnosed UC who demonstrated normal endoscopic findings at the time of enrollment were categorized as being in mucosal healing.^6^

For CD, patients were grouped according to the presence or absence of fistulizing complications. In addition to phenotypic classification, clinical disease activity was evaluated using the Pediatric Crohn’s Disease Activity Index (PCDAI).^12^ Furthermore, luminal endoscopic activity was quantified using the Simple Endoscopic Score for CD (SES-CD), which was calculated based on the ulcer size, ulcerated surface, affected surface, and presence of narrowing across 5 bowel segments.^13^

### Laboratory Measurements

The hemostatic profile included the assessment of factors V, VII, VIII, and X, vWF, prothrombin time (PT), activated partial thromboplastin time, proteins C and S, AT III, fibrinogen, and D-dimer levels. Inflammatory markers were also measured, including platelet count, erythrocyte sedimentation rate (ESR), C-reactive protein (CRP), and serum albumin.

### Sample Collection and Assay Procedures

Blood samples were collected in the morning after an overnight fast, during the same outpatient visit as the endoscopic evaluation, although not necessarily on the same day. To minimize confounding, patients with acute infections or severe exacerbations requiring hospitalization were excluded. Venous blood for coagulation assays was drawn into 3.2% sodium citrate tubes (9 : 1 blood-to-anticoagulant ratio), processed within minutes, and analyzed on the same day. Citrated plasma was prepared by double centrifugation at 2500 *g* for 15 minutes. Platelet counts were determined from ethylenediaminetetraacetic acid (EDTA)-anticoagulated blood.

Platelet counts, ESR, CRP, PT/international normalized ratio (INR), fibrinogen, coagulation factors (V, VII, VIII, and X), proteins C and S, and AT III were measured using validated automated hematological and coagulometric assays in the hospital’s central laboratory. All analyses were performed according to manufacturer’s instructions and internal quality control standards.

### Statistical Analysis

All statistical analyses were performed using IBM SPSS Statistics for Windows, version 28.0 (IBM SPSS Corp.; Armonk, NY, USA). The distribution of continuous variables was assessed using the Shapiro–Wilk test. Because most numerical data did not follow a normal distribution, nonparametric statistical methods were applied. Normally distributed continuous variables (e.g., age) are presented as mean ± SD, whereas non-normally distributed variables are presented as median and interquartile range (IQR). Categorical variables are expressed as counts and percentages. The Mann–Whitney *U*-test was used to compare 2 independent groups (e.g., corticosteroid users vs. nonusers), and the Kruskal–Wallis test was applied for comparisons across more than 2 groups, such as PUCAI-based disease activity categories. Correlations between coagulation parameters and inflammatory markers (CRP, ESR, and albumin), as well as disease activity indices including the PCDAI, SES-CD, and Total/Endoscopic Mayo Scores, were analyzed using the Spearman’s rank correlation coefficient (*ρ*) method. Categorical data were compared using the chi-square test or Fisher’s exact test, as appropriate.

All statistical tests were two-tailed, and a *P*-value < .05 was considered statistically significant. Due to the non-normal distribution of data and limited sample size, no formal correction for multiple comparisons (e.g., Bonferroni) was applied; therefore, the findings should be interpreted with appropriate caution.

## Results

### Patient Characteristics and Thrombosis Screening

A total of 46 patients with pediatric IBD were included (34 UC, 12 CD; mean age: 13.8 ± 2.6 years; 24 males). None of the patients had a history of clinical VTE. A chronic popliteal vein thrombus was incidentally detected in 1 patient with asymptomatic CD. The diagnosis was confirmed by Doppler ultrasonography, which revealed typical features of chronic thrombosis (echogenic material within the vein and absence of acute inflammatory changes), and no anticoagulation treatment was required. In the CD cohort, all patients exhibited inflammatory behaviors (Paris B1) without strictures, abscesses, or internal fistulas; fistulizing complications were exclusively perianal. Demographic and clinical characteristics of the study cohort, including medication use and disease activity scores, are presented in Table 1.

### Comparison of Coagulation Parameters Between Ulcerative Colitis and Crohn’s Disease

Factor VIII activity was higher in UC compared to CD (median [IQR]: 109.5 [87.95-173.65] vs. 74.3 [56.25-100.33]; *P* = .007; *r* = 0.40), suggesting a moderate effect size. Although vWF levels were lower in the CD group compared to UC (86.15 [53.45-105.95] vs. 109.0 [81.07-171.75]), the difference did not reach statistical significance (*P* = .064). Similarly, factors V, VII, and X, proteins C and S, antithrombin III, fibrinogen, and D-dimer showed no significant differences between the 2 groups (*P* > .05). Detailed hemostatic comparisons between UC and CD are presented in Supplementary Table 1.

### Coagulation Parameters by Disease Activity

**Figure 1.**
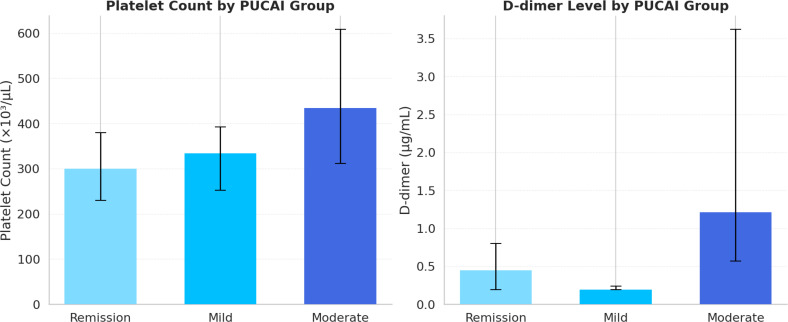
Platelet and D-dimer levels according to PUCAI-based clinical disease activity in ulcerative colitis. Bar plots show the mean ± SD of platelet counts and D-dimer levels across PUCAI-defined groups (remission n = 20, mild n = 9, moderate n = 5). Platelet counts and D-dimer levels significantly increased with clinical severity (*P* = .031 and *P* = .042, respectively).

In patients with UC, stratification by PUCAI scores revealed that both platelet count and D-dimer levels increased significantly in correlation with higher clinical disease activity (*P* = .031 and *P* = .042, respectively). Median platelet counts increased from 334.5 × 10^3^/μL (IQR: 252.75-392.75) in mild disease to 434.5 × 10^3^/μL (IQR: 311.5-608.25) in moderate disease, whereas D-dimer levels showed a similar increase, rising from 0.19 µg/mL (IQR: 0.19-0.24) to 1.21 µg/mL (IQR: 0.57-3.62) (Figure 1). When stratified by PUCAI scores, no significant differences were observed in the levels of fibrinogen, coagulation factors (V, VII, VIII, and X), vWF, or natural anticoagulants (proteins C and S and AT III) (all *P* > .05). In addition to clinical scoring, disease severity was evaluated using the Total Mayo Score (incorporating both clinical and endoscopic findings). Significant positive correlations with factor VIII (*r* = 0.487, *P* = .004) and AT III (*r* = 0.351, *P* = .042) were observed. However, the correlation with platelet counts did not reach statistical significance (*r* = 0.308, *P* = .076). To specifically isolate the impact of mucosal inflammation, the Endoscopic Mayo Score was further analyzed. This analysis confirmed the strong positive correlation with factor VIII (*r* = 0.482, *P* = .004) and, notably, revealed a significant correlation with platelet counts (*r* = 0.370, *P* = .031). Although D-dimer levels showed an increasing trend with endoscopic severity, this association did not reach statistical significance (*P* = .148).

In patients with CD, coagulation parameters did not differ significantly between those with and without fistulizing complications (all *P* > .05). Given that phenotype-based classification did not reflect hemostatic alterations, the disease activity was further evaluated using the PCDAI (clinical) score. Specifically, fibrinogen levels demonstrated a strong positive correlation with PCDAI scores (*r* = 0.692, *P* = .012), and factor VIII activity was significantly associated with clinical severity (*r* = 0.598, *P* = .040). Conversely, the endoscopic SES-CD score did not show statistically significant correlations in this subgroup, suggesting that systemic clinical indices (PCDAI) may be more reflective of the hemostatic burden in pediatric CD.

### Association Between Endoscopic Extent and Platelet Count in Ulcerative Colitis

**Figure 2.**
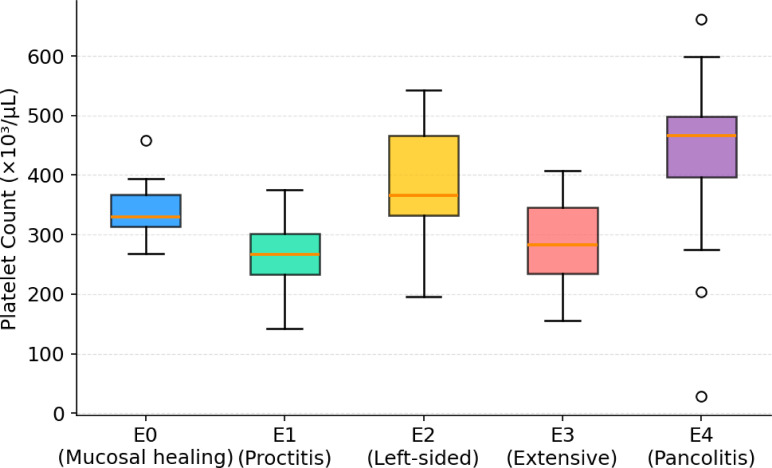
Platelet count by extent of endoscopic involvement in ulcerative colitis. Boxplot illustrates the distribution of platelet counts across different extents of endoscopic involvement in ulcerative colitis (E0 n = 6, E1 n = 12, E2 n = 5, E3 n = 5, E4 n = 8). An increasing trend in platelet count is observed from E0 to E4, consistent with a greater inflammatory burden. The upward trend was statistically significant (*P* = .008).

Platelet counts were significantly associated with the endoscopic disease extent in UC (Kruskal–Wallis *P* = .008). Median platelet values were 255 × 10^3^/µL (IQR: 228-364) in E1 (proctitis), 387 × 10^3^/µL (IQR: 337.8-492) in E2 (left-sided colitis), 303 × 10^3^/µL (IQR: 213-338) in E3 (extensive colitis), and 379 × 10^3^/µL (IQR: 301-528) in E4 (pancolitis). Post hoc analysis revealed that this significance was primarily driven by the difference between E1 and E4 subgroups (*P* < .05). Collectively, these findings suggested that broader mucosal involvement may be accompanied by increased thrombopoietic activity (Figure 2). In contrast, no significant associations were found between the endoscopic extent and other coagulation parameters (all *P* > .05).

### Correlation Between Inflammatory Markers and Coagulation Parameters

**Figure 3.**
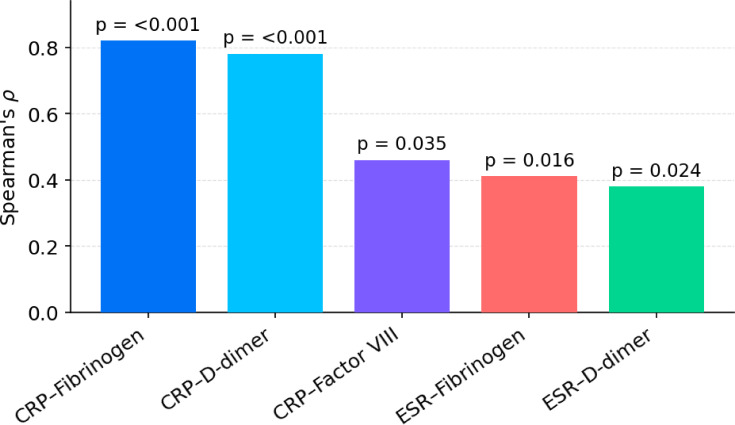
Correlation between inflammatory markers and coagulation parameters in UC. Bar heights represent Spearman’s correlation coefficients (*ρ*) between CRP/ESR and selected coagulation factors. *P*-values are displayed above each bar. Strong positive correlations were observed, particularly between CRP and fibrinogen/D-dimer levels.

In UC, CRP levels were positively correlated with fibrinogen (*ρ* = 0.823, *P* < .001), D-dimer (*ρ* = 0.785, *P* < .001), and factor VIII (*ρ* = 0.463, *P* = .035). Albumin demonstrated an inverse association with D-dimer (*ρ* = −0.585, *P* = .003). Erythrocyte sedimentation rate was also correlated with fibrinogen (*ρ* = 0.411, *P* = .016) and D-dimer (*ρ* = 0.388, *P* = .024). There were no significant correlations between inflammatory markers and other hemostatic parameters (all *P* > .05). These correlations are presented in Figure 3.

**Figure 4.**
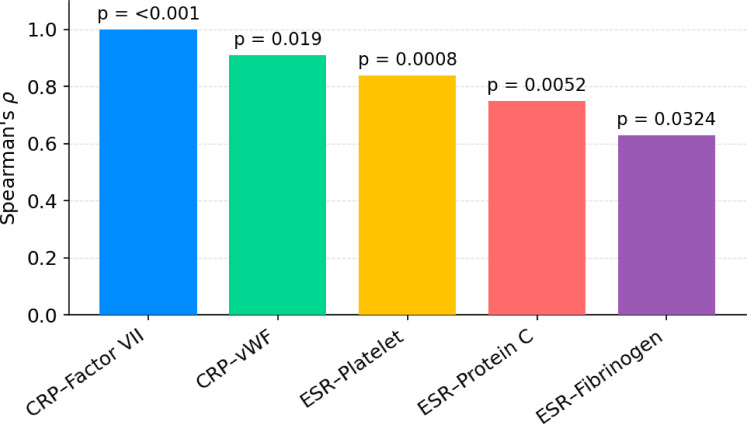
Correlation between inflammatory markers and coagulation parameters in Crohn’s disease. Spearman’s correlation coefficients (*ρ*) between CRP and ESR levels and selected coagulation parameters (factor VII, vWF, platelet count, protein C, and fibrinogen) are shown for patients with Crohn’s disease. *P*-values are indicated above each bar. Strong positive correlations were observed, particularly between CRP and factor VII and between ESR and platelet count.

In patients with CD, CRP revealed strong positive correlations with factor VII (*ρ* = 1.000, *P* < .001) and vWF (*ρ* = 0.886, *P* = .019). Erythrocyte sedimentation rate was positively correlated with platelet count (*ρ* = 0.832, *P* = .0008), protein C (*ρ* = 0.747, *P* = .005), and fibrinogen (*ρ* = 0.618, *P* = .032). However, because of the limited sample size—particularly in subgroup analyses—these correlations should be interpreted with caution and considered exploratory rather than definitive. The relevant associations are presented in Figure 4.

### Factor Levels According to Medication Use

**Figure 5.**
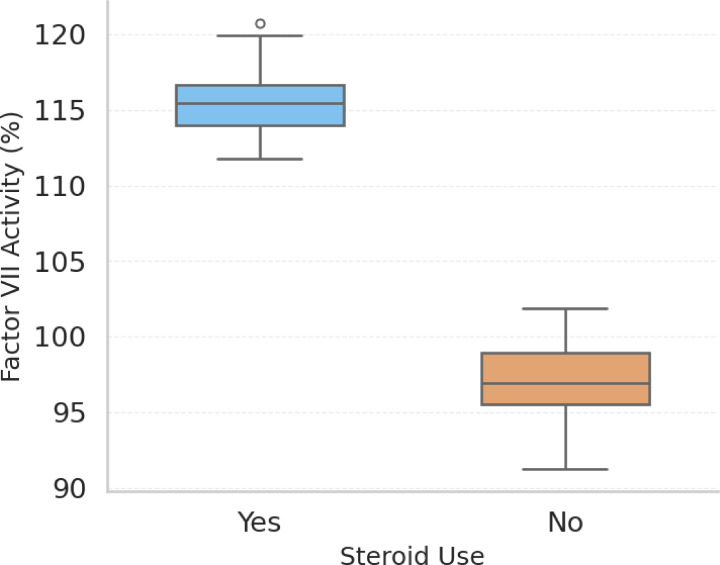
Comparison of factor VII activity (%) between patients with pediatric IBD receiving corticosteroids and those not receiving them. Median values, interquartile ranges, and outliers are shown. Factor VII activity was significantly higher in the steroid-treated group (*P* = .017).

Factor VII activity was significantly higher in patients receiving corticosteroid therapy compared to those not receiving steroids (median [IQR]: 117.0 [109.95-124.72] vs. 96.05 [84.35-105.45]; *U* = 193.5, *P* = .017, *r* = 0.35). However, given the small number of patients treated with steroids, this association should be interpreted cautiously and regarded as exploratory rather than confirmatory (Figure 5).

## Discussion

The current study assessed the prevalence of asymptomatic thrombosis and its associations with coagulation markers, systemic inflammation, and disease activity in pediatric IBD. Although thrombotic risk was low, platelet count, D-dimer, and factor VIII levels correlated with inflammatory burden, highlighting their potential utility in risk stratification and disease monitoring. Although similar associations between inflammatory burden and hemostatic alterations have been extensively reported in adult IBD, pediatric data remain limited. To the authors’ knowledge, this is the first study to concurrently assess clinical activity, endoscopic severity, and hemostatic profiles alongside Doppler ultrasonography in children. This gap in pediatric evidence has also been highlighted in the recent 2025 ESPGHAN–European Crohn's and Colitis Organisation (ECCO) guideline.^14^

In adults, IBD is associated with a nearly 3-fold increased VTE risk, particularly during active disease.^7^ In contrast, pediatric data remain limited and heterogeneous, lacking a standardized screening strategy. The recently published guidelines recommend considering pharmacological thromboprophylaxis in all inpatient children with acute severe colitis to reduce VTE risk while noting that decisions may be individualized in mobile young children (<12 years).^14^

Only 1 incidental chronic popliteal vein thrombus was detected in an asymptomatic patient with CD, a finding consistent with large-scale cohorts reporting a significantly lower VTE incidence in children (0.3%) compared to adults.^15^^,^^16^ However, given the limited sensitivity of Doppler ultrasonography for detecting pelvic or central thrombi, silent VTE may be underdiagnosed. Consequently, routine screening in asymptomatic children appears to offer low diagnostic yield, supporting a shift toward selective, risk-based surveillance.

The current study demonstrated that children with UC exhibited significantly higher coagulation factor VIII levels compared with those with CD. Proinflammatory cytokines, particularly interleukin-6 (IL-6), are known to enhance hepatic synthesis of factor VIII.^17^ Recent evidence also suggests that intestinal epithelial cells can produce factor VIII, particularly in the setting of continuous mucosal inflammation.^18^ Thus, elevated factor VIII in UC likely reflects both a stronger systemic acute-phase response and potentially increased local mucosal synthesis.

The significant positive correlation between platelet counts and the Endoscopic Mayo Score, coupled with the observation that platelet counts were significantly higher in patients with greater endoscopic disease extent (specifically pancolitis), links thrombocytosis directly to objective mucosal injury, rather than solely to systemic symptoms. Although IL-6 and thrombopoietin drive megakaryopoiesis, activated platelets also actively promote localized inflammation.^19^^,^^20^ Increased platelet levels likely reflect both a consequence and an amplifier of systemic inflammation, mirroring the severity of mucosal injury observed in the current study.^19^

Associations between D-dimer, fibrinogen, factor VIII, and inflammatory markers highlight the role of systemic inflammation in coagulation activation in UC. The strong correlation between CRP and factor VIII suggests that inflammation stimulates procoagulant pathways at both hepatic and endothelial levels. Interleukin-6 promotes fibrinogen production and endothelial release of factor VIII, contributing to a prothrombotic state.^21^ Furthermore, the inverse relationship between albumin and D-dimer reinforces this link. Collectively, these findings suggest that D-dimer, fibrinogen, and factor VIII serve as indicators of both thrombotic risk and inflammatory burden.

In UC, the significant correlation of Endoscopic Mayo Score with factor VIII and platelets indicates that hemostatic alterations parallel mucosal inflammation severity. In contrast, in patients with CD, clinical severity (PCDAI) correlated strongly with fibrinogen and factor VIII, whereas SES-CD did not, further supporting the hypothesis that systemic inflammatory burden may be the primary driver of coagulopathy in this subgroup. However, while these markers reliably track disease activity, their predictive value for thrombotic events requires prospective validation.

In adults with IBD, active disease is linked to increased levels of coagulation factors and fibrinogen, reduced proteins C and S, and AT III.^22^^,^^23^ However, the predictive value of these alterations for thrombotic risk remains inconsistent.^21^^,^^24^ In contrast, except for the relationship between factor VIII and the Mayo Score for UC and the relationship between PCDAI and factor VIII, as well as fibrinogen for CD, the pediatric cohort did not show similar alterations. This relative preservation of hemostatic balance regarding natural anticoagulants likely reflects the lower burden of vascular comorbidities in children compared to adults.

Corticosteroids promote a hypercoagulable state by increasing plasminogen activator inhibitor-1 (PAI-1) and impairing fibrinolysis.^25^ Consistent with this mechanism, significantly higher factor VII activity was observed in corticosteroid users. This aligns with prior evidence, indicating a 5-fold higher VTE risk in patients treated with corticosteroids.^26^ However, given the small sample size, this finding should be interpreted as exploratory.

A major strength of this study is the comprehensive evaluation of coagulation profiles alongside both clinical and endoscopic disease activity scores (Mayo Score and SES-CD) in a well-defined pediatric IBD cohort. Screening for asymptomatic thrombosis using Doppler ultrasonography during remission added robustness through objective, imaging-based confirmation.

This study has several limitations, primarily its small sample size and single-center design, which restrict generalizability. Although the absence of a healthy control group limits the interpretation of hemostatic elevations against normative values, consecutive enrollment was used to mitigate selection bias. Furthermore, because of the exploratory nature of the analysis, multiple comparisons or multivariable adjustments were not corrected; thus, the observed associations should not be interpreted as independent causal effects. Specifically, the lack of significant correlation between SES-CD and coagulation markers in the CD cohort likely reflects insufficient statistical power rather than the absence of a biological link. Future multicenter studies with larger cohorts and age-matched controls are required to validate these findings.

In conclusion, although asymptomatic thrombosis is rare in pediatric IBD, hemostatic markers—specifically factor VIII and platelets—closely mirror endoscopic severity in UC. Although these accessible parameters may serve as surrogate indicators of mucosal inflammatory burden and aid in risk stratification, they cannot be considered to eliminate the need for endoscopic evaluation. Given the low diagnostic yield of routine screening for silent thrombosis, a selective, risk-based approach is advisable. Future multicenter studies are required to validate these biomarkers and establish clinically relevant cut-off values.

## Supplementary Materials

Supplementary Material

## Figures and Tables

**Table 1.** Demographic and Clinical Characteristics of Pediatric Patients with Inflammatory Bowel Disease

**Characteristics**	**Ulcerative Colitis (n = 34)**	**Crohn’s Disease (n = 12)**
Male/female, n	14/20	10/2
Age, years (mean ± SD)	14.0 ± 2.7	13.6 ± 3.3
Medication use, n (%)		
Mesalazine	26 (76.5)	0 (0.0)
Azathioprine	21 (61.8)	7 (58.3)
Steroid	5 (14.7)	1 (8.3)
Infliximab	9 (26.5)	4 (33.3)
Disease activity, n		
Remission	20	7
Mild	9	1
Moderate	5	1
Severe	0	3

## Data Availability

The data that support the findings of this study are available on request from the corresponding author.

## References

[1] Klomberg RCW Hellendoorn AE Kemos P Rare and severe adverse events in children with inflammatory bowel disease: analysis of data from the PIBD-SETQuality Safety Registry. Lancet Child Adolesc Health. 2024;8(6):422 432. (doi: 10.1016/S2352-4642(24)00078-6) 38697175

[2] Kuenzig ME Bitton A Carroll MW Inflammatory bowel disease increases the risk of venous thromboembolism in children: a population-based matched cohort study. J Crohns Colitis. 2021;15(12):2031 2040. (doi: 10.1093/ecco-jcc/jjab113) 34175936 PMC8684458

[3] Magro F Soares JB Fernandes D. Venous thrombosis and prothrombotic factors in inflammatory bowel disease. World J Gastroenterol. 2014;20(17):4857 4872. (doi: 10.3748/wjg.v20.i17.4857) 24803797 PMC4009517

[4] Durak MB Cagin YF Balkan A Three-decade analysis of inflammatory bowel disease in Türkiye: a multicenter study (1993-2024). Turk J Gastroenterol. 2025;36(12):822-833. (doi: 10.5152/tjg.2025.25063) PMC12684269 41340405

[5] Gandhi J Mages K Kucine N Venous thromboembolism in pediatric inflammatory bowel disease: a scoping review. J Pediatr Gastroenterol Nutr. 2023;77(4):491 498. (doi: 10.1097/MPG.0000000000003889) 37455339

[6] Turner D Ruemmele FM Orlanski-Meyer E Management of paediatric ulcerative colitis, part 1: ambulatory care-an evidence-based guideline from European Crohn’s and Colitis Organization and European Society of Paediatric Gastroenterology, Hepatology and Nutrition. J Pediatr Gastroenterol Nutr. 2018;67(2):257 291. (doi: 10.1097/MPG.0000000000002035) 30044357

[7] Van Rheenen PF Aloi M Assa A The medical management of paediatric Crohn’s disease: an ECCO-ESPGHAN guideline update. J Crohns Colitis. 2021;15(2):jjaa161. (doi: 10.1093/ecco-jcc/jjaa161) 33026087

[8] Levine A Koletzko S Turner D ESPGHAN revised porto criteria for the diagnosis of inflammatory bowel disease in children and adolescents. J Pediatr Gastroenterol Nutr. 2014;58(6):795 806. (doi: 10.1097/MPG.0000000000000239) 24231644

[9] Turner D Otley AR Mack D Development, validation, and evaluation of a pediatric ulcerative colitis activity index: a prospective multicenter study. Gastroenterology. 2007;133(2):423 432. (doi: 10.1053/j.gastro.2007.05.029) 17681163

[10] Lewis JD Chuai S Nessel L Use of the noninvasive components of the Mayo score to assess clinical response in ulcerative colitis. Inflamm Bowel Dis. 2008;14(12):1660 1666. (doi: 10.1002/ibd.20520) 18623174 PMC2597552

[11] Levine A Griffiths A Markowitz J Pediatric modification of the Montreal classification for inflammatory bowel disease: the Paris classification. Inflamm Bowel Dis. 2011;17(6):1314 1321. (doi: 10.1002/ibd.21493) 21560194

[12] Hyams JS Ferry GD Mandel FS Development and validation of a pediatric Crohn’s disease activity index. J Pediatr Gastroenterol Nutr. 1991;12(4):439 447. (doi: 10.1097/00005176-199105000-00005) 1678008

[13] Daperno M D’Haens G Van Assche G Development and validation of a new, simplified endoscopic activity score for Crohn’s disease: the SES-CD. Gastrointest Endosc. 2004;60(4):505 512. (doi: 10.1016/S0016-5107(04)01878-4) 15472670

[14] Assa A Aloi M Van Biervliet S Management of paediatric ulcerative colitis, part 2: acute severe colitis-an updated evidence-based consensus guideline from the European Society of Paediatric Gastroenterology, Hepatology and Nutrition and the European Crohn’s and Colitis Organization. J Pediatr Gastroenterol Nutr. 2025;81(3):816 851. (doi: 10.1002/jpn3.70096) 40528309

[15] De Laffolie J Ballauff A Wirth S Occurrence of thromboembolism in paediatric patients with inflammatory bowel disease: data from the CEDATA-GPGE registry. Front Pediatr. 2022;10:883183. (doi: 10.3389/fped.2022.883183) PMC9204097 35722497

[16] Nguyen GC Wu H Gulamhusein A The utility of screening for asymptomatic lower extremity deep venous thrombosis during inflammatory bowel disease flares: a pilot study. Inflamm Bowel Dis. 2013;19(5):1053 1058. (doi: 10.1097/MIB.0b013e3182802a65) 23429463

[17] Bittar LF Mazetto B Orsi FLA Long-term increased factor VIII levels are associated to interleukin-6 levels but not to post-thrombotic syndrome in patients with deep venous thrombosis. Thromb Res. 2015;135(3):497 501. (doi: 10.1016/j.thromres.2014.12.024) 25575413

[18] Pan J Dymond C Hopman W Intestinal epithelial cells synthesize and secrete factor VIII in vitro and in vivo. Blood. 2016;128(1):108 118. (doi: 10.1182/blood-2015-12-687251)

[19] Couldwell G Machlus KR. Modulation of megakaryopoiesis and platelet production during inflammation. Thromb Res. 2019;179:114 120. (doi: 10.1016/j.thromres.2019.05.008) 31128560

[20] Koupenova M Clancy L Corkrey HA Circulating platelets as mediators of immunity, inflammation, and thrombosis. Circ Res. 2018;122(2):337 351. (doi: 10.1161/CIRCRESAHA.117.310795) 29348254 PMC5777300

[21] Lagrange J Lacolley P Wahl D Shedding light on hemostasis in patients with inflammatory bowel diseases. Clin Gastroenterol Hepatol. 2021;19(6):1088 1097.e6. (doi: 10.1016/j.cgh.2019.12.043) 31972287

[22] Owczarek D Cibor D Głowacki MK Inflammatory bowel disease: epidemiology, pathology and risk factors for hypercoagulability. World J Gastroenterol. 2014;20(1):53 63. (doi: 10.3748/wjg.v20.i1.53) 24415858 PMC3886032

[23] Cakal B Gokmen A Yalinkilic M Natural anticoagulant protein levels in Turkish patients with inflammatory bowel disease. Blood Coagul Fibrinolysis. 2010;21(2):118 121. (doi: 10.1097/MBC.0b013e328335d025) 20040858

[24] Bosch A Brunsvig Jarvis KB Brandão LR The role of coagulation factors VIII, IX and XI in the prediction and mediation of recurrent thrombotic events in children with non-central venous catheter deep vein thrombosis. Thromb Res. 2024;236:228 235. (doi: 10.1016/j.thromres.2024.03.009) 38484629

[25] Alkim H Koksal AR Boga S Etiopathogenesis, prevention, and treatment of thromboembolism in inflammatory bowel disease. Clin Appl Thromb Hemost. 2017;23(6):501 510. (doi: 10.1177/1076029616632906) 26893444

[26] Higgins PDR Skup M Mulani PM Increased risk of venous thromboembolic events with corticosteroid vs biologic therapy for inflammatory bowel disease. Clin Gastroenterol Hepatol. 2015;13(2):316 321. (doi: 10.1016/j.cgh.2014.07.017) 25038374

