# Supplementary material for: Thrombotic Risk and Hemostatic Profiles in Pediatric Inflammatory Bowel Disease: Association with Disease Activity
Source: Turk J Gastroenterol. 2026 Mar 24;37(6):657–64. doi: 10.5152/tjg.2026.25541 (PMC13247858; doi:10.5152/tjg.2026.25541)
Supplement: Supplementary Material [file supplementary_material.pdf]

Supplementary Table 1. Comparison of hemostatic parameters between pediatric patients with ulcerative colitis and Crohn's disease

Parameter	Ulcerative Colitis, median (IQR) (n = 34)	Crohn's Disease, median (IQR) (n = 12)	*P*
Factor V	89.65 (72.10-111.55)	97.75 (79.88-120.50)	.764
Factor VII	99.10 (87.33-109.65)	95.25 (83.00-111.30)	.626
Factor VIII	109.50 (87.95-173.65)	74.30 (56.25-100.33)	**.007***
Factor X	106.50 (88.90-119.60)	105.50 (96.88-120.67)	.717
vWF	109.00 (81.07-171.75)	86.15 (53.45-105.95)	.064
Protein C	90.40 (77.65-100.40)	96.85 (83.95-112.28)	.451
Protein S	80.60 (69.17-93.78)	87.10 (78.93-100.15)	.634
Antithrombin III	110.85 (103.83-116.60)	111.70 (109.22-116.47)	.476
D-dimer	0.19 (0.19-0.27)	0.19 (0.19-0.21)	.452
Fibrinogen	294.00 (258.50-331.50)	280.50 (245.50-363.50)	.783

Values are presented as median (IQR). Statistically significant results ($P < .05$) are shown in bold. Coagulation factors and anticoagulants are reported as % activity; fibrinogen in mg/dL, D-dimer in μg/mL FEU.
